# The effects of behavioral intervention on anthropometric, clinical, and biochemical parameters in patients with polycystic ovary syndrome: a systematic review and meta-analysis

**DOI:** 10.3389/fendo.2024.1297841

**Published:** 2024-02-28

**Authors:** Min Xie, Yang Yang, Jing Zhang

**Affiliations:** ^1^ Department of Obstetrics and Gynecology, West China Second University Hospital, Sichuan University, Chengdu, China; ^2^ Key Laboratory of Birth Defects and Related Diseases of Women and Children (Sichuan University), Ministry of Education, Chengdu, China; ^3^ Reproductive Endocrinology and Regulation Laboratory, West China Second University Hospital, Sichuan University, Chengdu, China; ^4^ The Joint Laboratory for Reproductive Medicine of Sichuan University, The Chinese University of Hong Kong, Chengdu, China; ^5^ Department of Obstetrics and Gynecology, Chengdu Qingbaijiang District People’s Hospital, Chengdu, China

**Keywords:** behavioral intervention, polycystic ovary syndrome, weight loss, body mass index, waist circumference, meta-analysis

## Abstract

**Objective:**

To evaluate the effects of behavioral intervention for polycystic ovary syndrome (PCOS).

**Methods:**

Electronic databases were searched, including Pubmed, Medline, EMBASE, and the Cochrane Central Register of Controlled Trials from inception to 1 April 2023. Inclusion criteria for this study required a diagnosis of PCOS. Interventions of interest included behavioral intervention and routine treatment compared with routine treatment. The studies included in the analysis were designed as randomized controlled trials (RCTs). We conducted meta-analyses following the recommended guidelines. The data was analyzed using either the random effects model or fixed effects model. The results of the studies were expressed as either mean differences (MD) or standardized mean differences (SMD) along with their corresponding 95% confidence intervals (CIs).

**Results:**

Eight RCTs were identified, including data from 744 patients (415 in the intervention group and 329 in the control group). The results indicate an improvement in the effectiveness of behavioral interventions for weight loss (MD: -1.07; 95% CI: -2.1 to 0.03; I^2^ = 0%; P=0.04), body mass index (BMI) (MD: -1.12; 95% CI: -1.92 to -0.33; I^2^ = 73%; P=0.006), waist circumference (MD: -3.97; 95% CI: -5.64 to -2.29; I^2^ = 0%; P<0.00001), quality of life about weight (MD: 0.58; 95% CI: 0.15 to 1.02; I^2^ = 0%; P=0.008), depression (SMD: -1.12; 95% CI: -2.35 to -0.07; I^2^ = 92%; P=0.04), and triglycerides (MD: -0.16; 95% CI: -0.27 to -0.05; I^2^ = 27%; P=0.004). However, there were no significant differences in menstrual cycles, hirsutism, emotions, and infertility. The study also found that behavioral interventions had no significant effect on systolic and diastolic blood pressure, high-density lipoprotein, low-density lipoprotein, homeostasis model assessment of insulin resistance, testosterone, total cholesterol, fasting glucose, fasting insulin, hemoglobin A1C, and sex hormone binding globulin.

**Conclusion:**

Behavioral intervention supplementation contributes to weight loss, reduction in BMI and waist circumference, and improvement in depression among patients with PCOS. However, no significant improvement was observed in the biochemical index and quality of life. The long-term effects of behavioral intervention for PCOS remain unclear due to limitations in the quality of the studies involved and the short duration of treatment.

**Systematic Review Registration:**

https://www.crd.york.ac.uk/PROSPERO, identifier CRD42023442875.

## 1 Introduction

Polycystic ovary syndrome (PCOS) is a common endocrine disorder that affects women of reproductive age (1). The estimated prevalence of PCOS worldwide is reported to be between 10% and 20% (2). Additionally, many cases remain undiagnosed (3). The main characteristics of PCOS include infrequent or absent menstrual periods, infertility, high levels of androgens (male hormones), excessive hair growth, obesity, and insulin resistance (4). The exact cause and pathogenesis of PCOS are still not yet fully understood, and there is currently no definitive cure for the condition (5). PCOS can significantly impact patients’ quality of life and lead to psychological issues such as low self-esteem and depression (6). PCOS patients often experience a range of symptoms, including menstrual and ovulation disorders, infertility, metabolic syndrome, emotional distress, and reproductive problems. Additionally, many PCOS patients are overweight or obese (7). The metabolic and reproductive characteristics of PCOS tend to deteriorate with obesity (8).

PCOS, as a complex multisystem disorder, has traditionally been treated with medications and surgeries. However, recent research has led to recognition and attention to treatment strategies such as lifestyle modification, psychological evaluation and interventions, long-term medication management, and multidisciplinary collaboration (9). Lifestyle interventions play a crucial role in the treatment of PCOS and are recommended as a first-line strategy to improve the health outcomes of PCOS patients (9). Current lifestyle recommendations focus on eating a healthy diet and regular physical activity (10). Adherence to these recommendations often requires major behavioral pattern change (11). Behavioral interventions are commonly employed to modify behavioral patterns (12), These interventions typically include text messages, mobile health applications, cognitive-behavioral interventions, supervised training, encouragement courses, psychoeducational group programs, psychological care, motivational interviewing, peer support, and educational group meetings (13, 14). Behavioral intervention has been successfully used in the treatment of various diseases, including obesity, coronary heart disease, psychological health, and depression (13, 15–17). Currently, behavioral intervention has been applied and reported as a treatment option for PCOS patients (18). However, there is inconsistent evidence regarding the effects of behavioral interventions on body composition, clinical manifestations, and biochemical indicators in patients with PCOS (19–32). Some studies suggest that behavioral interventions can lead to increased improvements in PCOS patients (20–28, 30, 31), while others have proved that behavioral interventions have no significant effect (29, 32). Additionally, the sample sizes of these studies have been relatively small.

Previous systematic reviews have primarily focused on the effectiveness and safety of lifestyle modifications, physical activity, and cognitive-behavioral interventions for patients with PCOS (33–35). However, limited attention has been given to behavioral interventions. Therefore, the main objective of this study is to conduct a systematic review and meta-analysis of published RCTs to comprehensively assess the positive effects of behavioral interventions on PCOS. By doing so, we aim to provide evidence-based recommendations for the treatment of patients with PCOS.

## 2 Methods

The present systematic review and meta-analysis was conducted with the Preferred Reporting Items for Systematic Reviews and Meta-Analyses (PRISMA) guidelines (36). The research focuses on the PICOS question: What are the effects of behavioral interventions on anthropometric measurements (weight loss, BMI, waist circumference), clinical outcomes (quality of life, psychological status), and biochemical indexes (high-density lipoprotein (HDL), low-density lipoprotein (LDL), blood pressure (BP), homeostasis model assessment of insulin resistance (HOMA-IR), testosterone (T), total cholesterol (Tch), triglycerides (TG), fasting glucose, fasting insulin, sex hormone binding globulin (SHBG), and hemoglobin A1C (HbA_1C_) in women with PCOS compared with conventional treatment after four weeks to 12 months of intervention? Prior to data extraction, the systematic review was registered in the PROSPERO database (CRD42023442875). Due to the lack of data, the minimum number of studies for the meta-analysis was decreased to two.

### 2.1 Inclusion criteria

Studies were included in the systematic review if they met the following criteria: (1) Participants: women with a definite diagnosis of PCOS; (2) Intervention: behavioral interventions, such as text messages, mobile health applications, cognitive-behavioral interventions, supervised training, encouragement courses, psychoeducational group programs, psychological care, motivational interviewing, peer support, and educational group meetings, compared with routine treatment without any behavioral intervention.; (3) Outcomes: outcomes included anthropometric measurements, clinical measures or biochemical markers, at least one of following statistics: weight loss, BMI, waist circumference, quality of life, psychological status, BP, HDL, LDL, HOMA-IR, T, Tch, TG, fasting glucose, fasting insulin, HbA1C, and SHBG; (4) Study designs: randomized controlled trials (RCTs).

### 2.2 Exclusion criteria

Excluded from the analysis were conference summaries, animal experiments, cohort studies, retrospective studies, non-randomized controlled intervention studies, studies with overlapping data, studies with unavailable full text or data, studies with unreported target outcomes, and non-English language literature.

### 2.3 Outcome indexes

The study’s primary outcome indicators were weight loss, BMI, and waist circumference. These outcomes are essential for evaluating the effectiveness of interventions or treatments aimed at improving clinical symptoms in patients with PCOS. BMI is a standardized measure that consider both weight and height, enabling a more standardized assessment of a patient’s body composition. The study’s secondary outcome measures in this study aim to provide a more comprehensive understanding of the effects of the condition on a patient’s life. These secondary outcome measures encompass clinical manifestations of PCOS, such as quality of life, psychological status, and blood pressure. The quality of life of patients with PCOS is evaluated using the disease-specific polycystic ovary syndrome questionnaire (PCOSQ) (37). The questionnaire comprises 26 items that measure five areas: emotions, hirsutism, weight, infertility problems, and menstrual problems. It enables researchers to investigate the effects of PCOS on a patient’s emotional well-being, self-image, fertility, and menstrual regularity. Furthermore, the study aims to examine various metabolic indicators associated with PCOS, including HOMA-IR, T, TG, LDL, HDL, Tch, fasting glucose, fasting insulin, HbA_1C_, and SHBG. Assessing these metabolic indicators can provide insights into the hormonal and metabolic dysregulation commonly observed in patients with PCOS, enabling a better understanding of the condition’s underlying mechanisms. This study aims to comprehensively evaluate the effectiveness of interventions or treatments for PCOS patients by incorporating both primary and secondary outcome measures. The evaluation will consider physical aspects such as weight loss and body composition, as well as psychological well-being and metabolic health.

### 2.4 Search strategies

The systematic search was performed in Pubmed, MEDLINE, EMBASE, and Cochrane Central Register of Controlled Trials (CENTRAL) from inception until April 1, 2023. We applied: “polycystic ovary syndrome” “PCOS” “polycystic ovarian syndrome” “behavioral therapy” “behavioral modification” “behavioral intervention” “behavior change interventions” “Randomized Controlled Trial” as search terms. The search strategy is available in **Supplementary Information**. Only English language studies were considered, and human filters were applied. Potential eligible studies were manually searched for additional data by reviewing relevant conference proceedings and reference lists.

### 2.5 Data extraction

**Figure 1:**
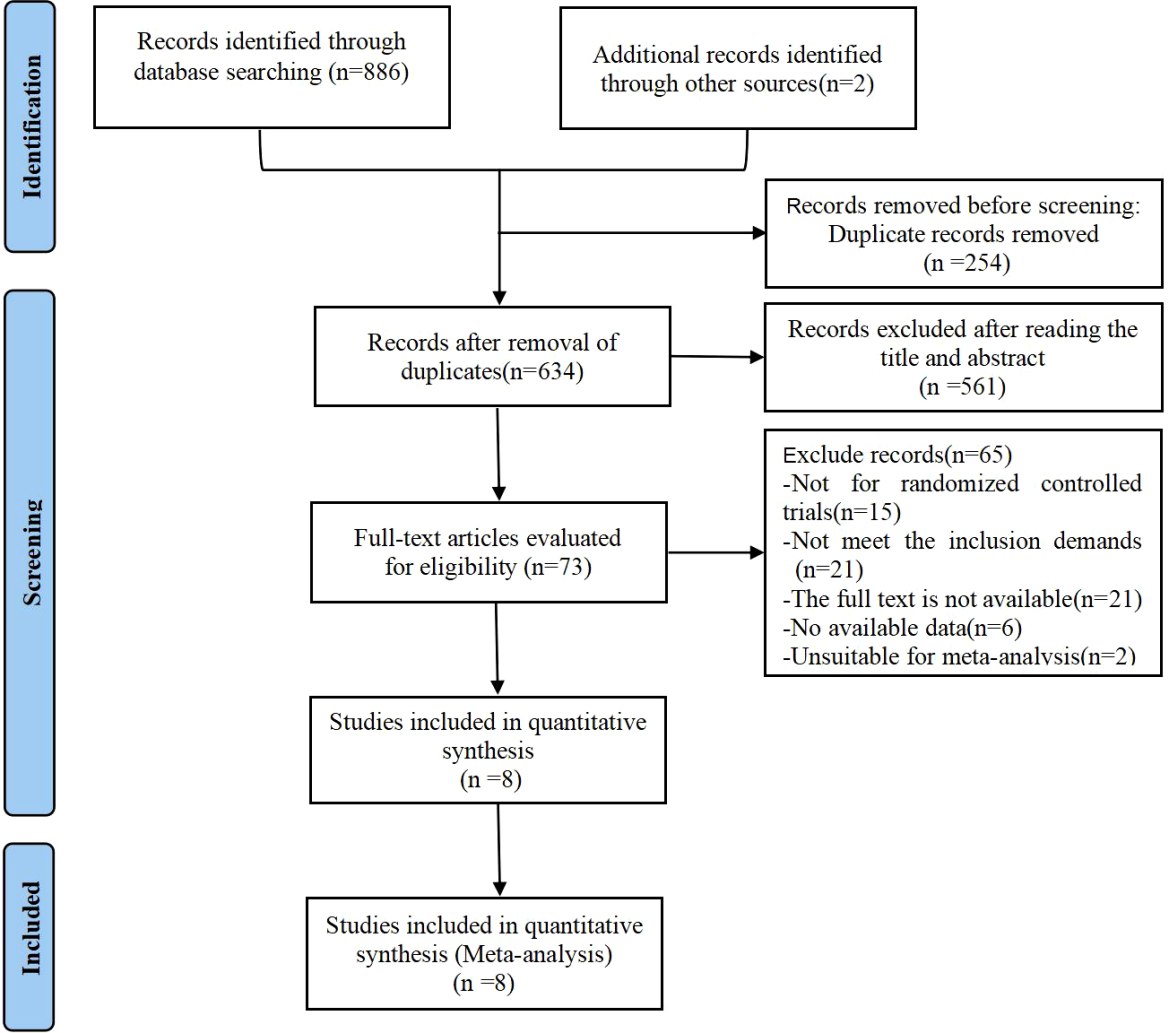
PRISMA flow diagram of the study process. PRISMA, Preferred Reporting Items for Systematic review and Meta- analysis.

Using Zotero to remove duplicate studies from the identified articles. Two review authors independently collected information and screened the abstracts. Full texts were retrieved for further analysis. The characteristics of included studies were extracted according to Cochrane guidelines by two authors. Inconsistencies were resolved through discussion with the third author. The selection process was documented with a flowchart of PRISMA (**Figure 1**).

A data collection sheet was standardized based on the consensus of clinical and methodological experts. Two review authors independently collected the following data: basic characteristics such as author, year, and country; subject characteristics including age and number of people in each group; and outcome indicators such as weight loss, BMI, waist circumference, quality of life, psychological status, HDL, LDL, HOMA-IR, T, Tch, TG, BP, fasting glucose, fasting insulin, HbA_1C_, and SHBG (**Table 1**). For continuous variables, we extracted mean and standard deviation (SD) values at baseline and after treatment. We sent emails to study authors for more detailed information and outcome data.

**Table 1:** Characteristics of the included studies.

Study	Country region	Publish year	Recruitment	Population				Sample size	Intervention	Control	Duration	Outcomes
				Mean age (y)		BMI						
Author				Intervention	Control	Intervention	Control					
**Abdollahi et** **al (** 25 **).**	**Iran**	**2018**	**2012.4-2015.1**	**28.0 (4.4)**	**27.0 (4.6)**	**27.6 (5.9)**	**29.2 (4.9)**	**74**	**cognitive behavioral therapy (encouragement courses)**	**routine treatments**	**4w**	②⑩
**Wang et al. (** 26)	**china**	**2022**	**2008.10-2010.3**	**24.72 (4.20)**	**24.94 (4.31)**	**25.99 (3.87)**	**25.25** **(3.95)**	**122**	**Transtheoretical model-based mobile health application**	**routine treatments**	**12m**	②③⑩
**Oberg et al. (** 29)	**Sweden**	**2019**	**2012.4-2015.1**	**31.0 (5.1)**	**29.9 (5.7)**	**33.5 (5.13)**	**34.3 (4.93)**	**68**	**behavioral modification intervention (encouragement courses)**	**routine treatments**	**12m**	①②③⑤⑪⑫⑭
**Legro et al. (** 30)	**Pennsylvania**	**2015**	**2008.10-2010.3**	**28.6 (3.77)**	**29.8 (3.7)**	**35.3 (4.5)**	**35.1 (4.2)**	**149**	**lifestyle modification and/or oral contraceptive pills (encouragement courses)**	**routine treatments**	**16w**	③④⑧⑪
**Benham et al. (** 31)	**Canada**	**2020**	**2017.12-2019.9**	**29.3 (4.3)**	**29.1 (5.4)**	**31.35 (8.6)**	**31.6 (8.2)**	**47**	**encourage supervision training**	**routine treatments**	**6m**	①②③⑤⑥⑦⑧⑨⑫⑬
**Guo et al. (** 27)	**China**	**2022**	**2021.3-2021.6**	**24.95 (4.02)**	**25.98 (4.05)**	**25.86 (2.64)**	**25.45 (2.42)**	**80**	**Transtheoretical model based intervention**	**routine treatments**	**6m**	②③
**Thomson et al. (** 32)	**Australia**	**2016**	**2006.4-2007.2**	**30.3 (6.2)**	**30.3 (6.2)**	**36.4 (5.6)**	**36.4 (5.6)**	**43**	**encourage supervision training**	**routine treatments**	**20w**	①④⑩
**Mani et al. (** 28)	**UK: United Kingdom**	**2018**	**2012.7-2013.7**	**33.4 (7.1)**	**33.3 (8.1)**	**34.2 (7.2)**	**33.2 (6.2)**	**161**	**A structured education program**	**routine treatments**	**12m**	①②④⑤⑥⑦⑧⑨⑪⑫⑬⑭

outcomes, ①weight loss; ②BMI, body mass index; ③waist circumference; ④PCOSQ, polycystic ovary syndrome questionnaire; ⑤HOMA-IR, homeostasis model assessment of insulin resistance; ⑥HDL/LDL, high-density lipoprotein/low-density lipoprotein; ⑦TG, triglycerides; ⑧Tch, total cholesterol; ⑨BP, blood pressure; ⑩depression; ⑪T, testosterone; ⑫fasting glucose/fasting insulin; ⑬HbA1C, hemoglobin A1C, ⑭SHBG, sex hormone binding globulin.

### 2.6 Data synthesis and analysis

All related statistical analysis was conducted by using the software Review Manager 5.4. MD was applied for continuous data such as weight loss and BMI. Standardized mean difference (SMD) was commonly used when different scales were taken for the same outcome. If MD was not mentioned, it was derived from either the standard error, interquartile range, or the 95% confidence interval. The review expressed effect sizes for each outcome measure as the weighted mean difference (WMD) and 95% CI between the behavioral interventions and routine treatment controls. Heterogeneity between studies was evaluated using I^2^ statistical analysis (I^2^ statistics>75% assigned as highly heterogeneous) and 95% confidence interval. The fixed effects model was applied when I²<50%; otherwise, the random effects model was used for further data analysis. Subgroup and sensitivity analyses were used to explore sources of heterogeneity. If necessary, we utilized Engauge Digitizer 4.1 to extract data from images. Publication bias was assessed using Begg’s and Egger’s tests when more than ten trials were included in the analysis.

### 2.7 Assessment of risk of bias and evidence quality

The risk of bias of the included studies was conducted independently by two authors using the Cochrane Collaboration’s tools and criteria (38). The domains typically evaluated using the Cochrane Collaboration’s risk of bias tool include sequence generation, allocation concealment, blinding, incomplete outcome data, selective outcome reporting, and other sources of bias, with the risk of bias for each domain classified as low, high, or unclear. Disagreements between data extractors were resolved by discussion with a third author.

## 3 Results

### 3.1 Studies selection and the flow chart

A total of 888 articles were retrieved from various databases, including 292 from PubMed, 139 from Embase, 264 from Cochrane library, 191 from Medline, and 2 from the references. After screening by Zotero, 634 articles were left after removing duplicates (n=254). Among these, 561 articles were excluded after screening of the titles and abstracts for irrelevance. Seventy-three articles were selected for full-text revision, and sixty-five of these were excluded for following reasons: (1) Not for randomized controlled trials(n=15); (2) Not meet the inclusion demands(n=21); (3) The full text is not available (unpublish trails or unable to find full text) (n=21); (4) No available data(n=6); (5) Unsuitable for meta-analysis(n=2). Finally, this systematic review analyzed eight RCTs with 744 patients with PCOS, as shown in **Figure 1** (25–32).

### 3.2 Characteristics of included studies

The basic characteristics of the eight RCTs including 744 subjects were listed in **Table 1**. The mean age of control group was 29.1 ± 6.4 years, while the behavioral intervention group was 29.9 ± 5.8 years. There was no significant difference in the age of the study participants. The mean baseline BMI was 31.0 ± 6.4 kg/m^2^ in the control groupand 31.8 ± 6.7 kg/m^2^ in the intervention group. There was no significant difference in the baseline BMI between the two groups of subjects.

These eight studies were published between 2015 and 2022. Two of the studies were performed in China (26, 27), and the remaining six were performed in Iran (25), Sweden (29), the United Kingdom (28), Pennsylvania (30), Canada (31), and Australia (32), respectively. Two studies evaluated the effectiveness of behavioral interventions using mobile health applications (26, 27). These interventions aimed to modify individuals’ behavior patterns in order to achieve positive health outcomes. Additionally, four studies evaluated the impact of encouragement courses as a behavioral intervention (25, 26, 29, 32). These interventions focused on patients participating in team-based courses, where healthcare professionals provided health education and behavior guidance to promote behavior change and improve overall health outcomes. Furthermore, two studies utilized supervised encouragement training as the central approach to modify behavior patterns and ultimately achieve the desired outcomes (30, 31). The aim of these interventions was to encourage individuals to adopt regular exercise routines to promote healthy behavior change. The studies had a follow-up duration ranging from four weeks to 12 months, allowing for longitudinal assessment and monitoring of participants’ progress in changing their behavior patterns. Regarding outcome indicators, four studies reported weight loss as a measurable outcome of the behavioral interventions. Additionally, six studies used BMI as the outcome measure, while four studies used waist circumference. Some studies also measured secondary outcomes such as quality of life, psychological status, HDL, LDL, HOMA-IR, T, Tch, TG, BP, fasting glucose, fasting insulin, HbA_1C_, and SHBG (**Table 1**).

### 3.3 Risk bias in included studies

**Figure 2:**
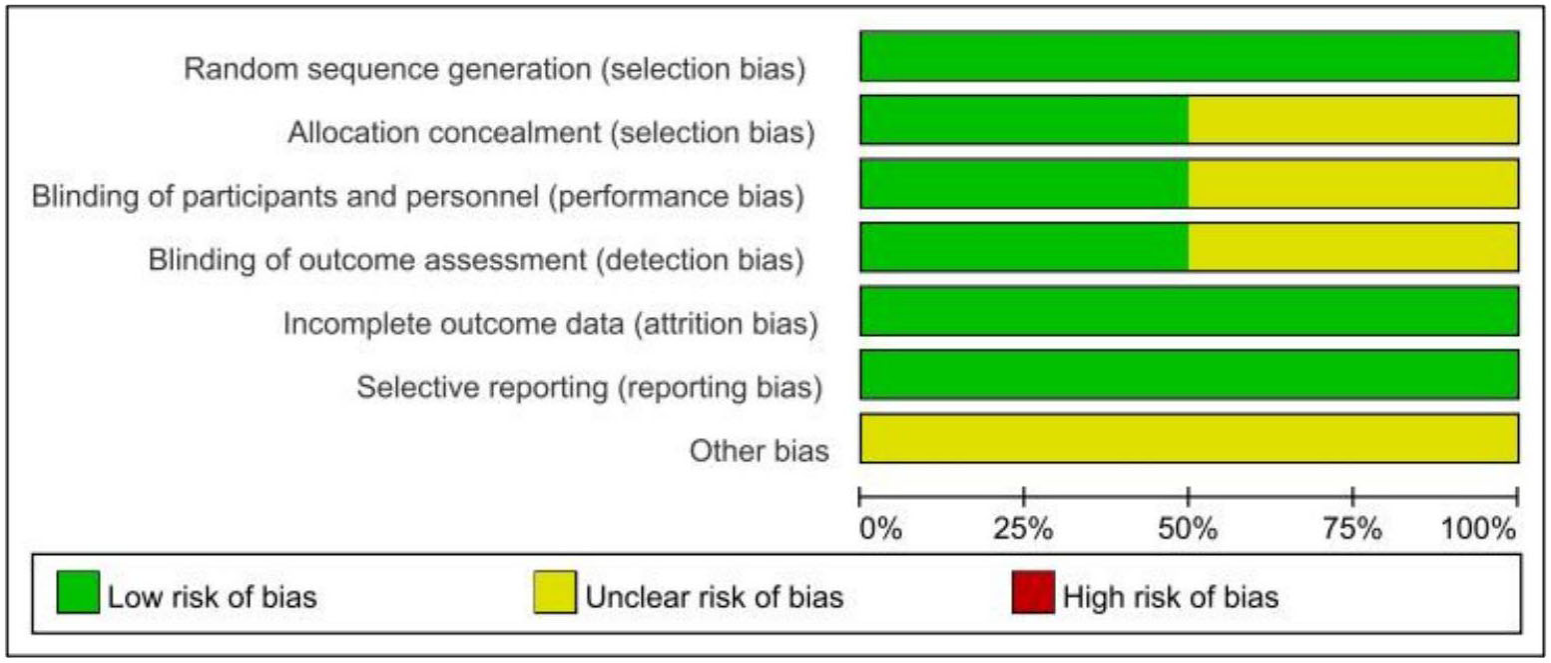
Overall risk of bias assessment.

**Figure 3:**
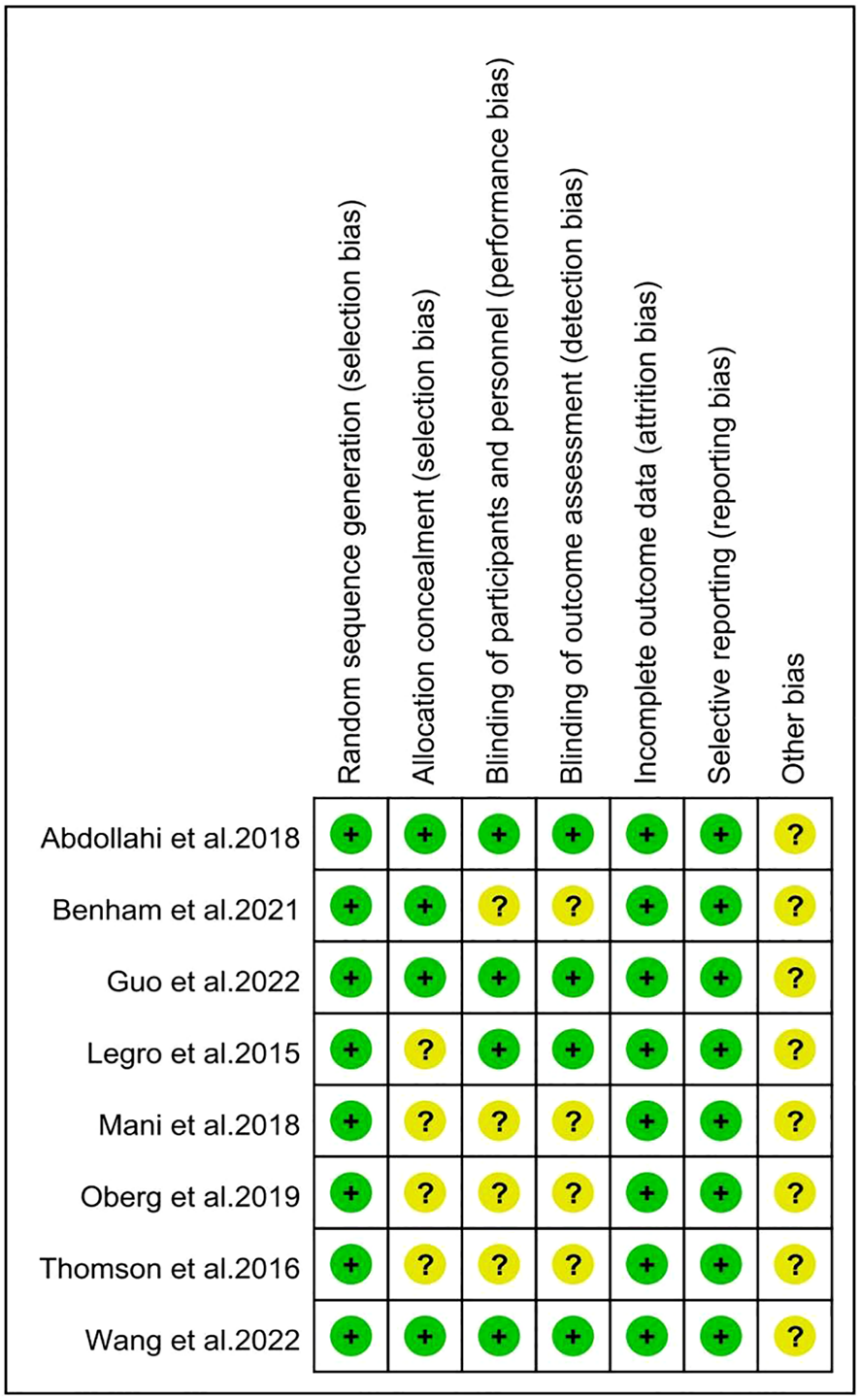
Risk of bias summary for individual studies. The symbol '?' indicates unclear risk of bias, while the symbol '+' indicates low risk of bias.

The risk of bias in the included studies was summarized in **Figures 2**, **3** according to the Cochrane risk of bias tool. All included trials reported adequate randomized sequence generation. Two studies used random number tables (30, 32), five studies used block randomization (25–27, 29, 31), and one study only mentioned randomization but did not provide further details (30). Four trials were assessed as being at unclear risk of selection bias because allocation concealment details were not provided (28–30, 32). Four trials were assessed as having an unclear risk of performance bias because they did not require blinding of participants or researchers (28, 29, 31, 32). Similarly, four studies mentioned the blinding of outcome assessors (25–27, 30), but the other four studies did not mention whether blinding of outcome assessors was carried out. All trials were preregistered in a clinical trial registry, which might have efficiently controlled reporting bias. Furthermore, these studies did not offer precise information regarding the existence of other potential sources of bias.

### 3.4 Data synthesis and meta-analysis

#### 3.4.1 Primary outcomes

##### 3.4.1.1 Weight loss

**Figure 4:**
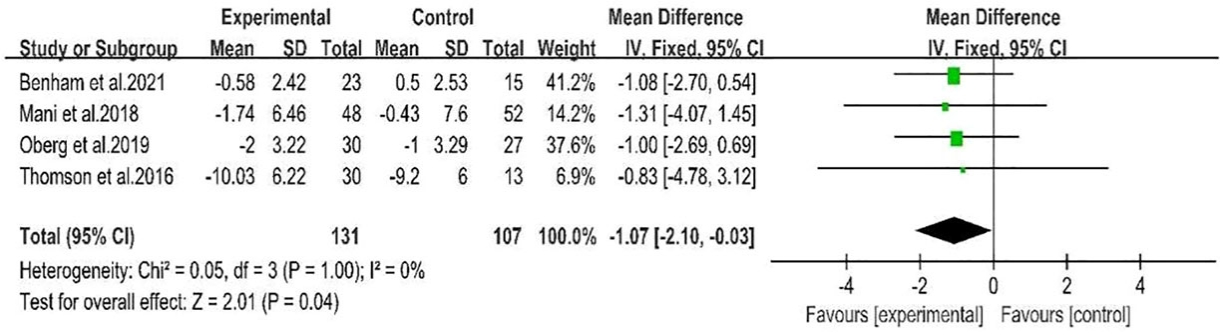
Forest plot of mean difference standardized of range of weight loss.

**Figure 5:**
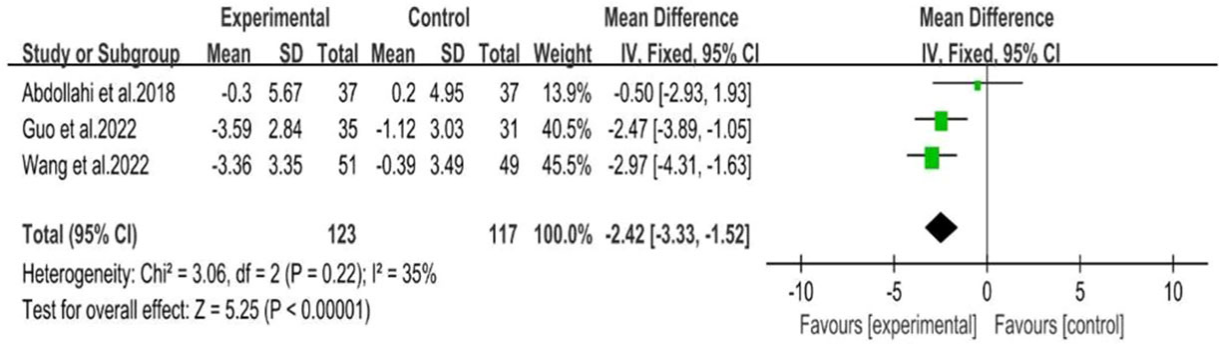
Forest plot of the BMI (the studies not mentioned weight loss).

Four studies (28, 29, 31, 32)showed a range of weight loss with 131 subjects in the behavioral intervention group and 107 subjects in the control group. The meta-analysis of RCTs using fixed effects models revealed that behavioral interventions were significantly more effective in reducing weight in patients with PCOS compared to the controls (MD: -1.07; 95% CI: -2.1 to -0.03; I^2^ = 0%; P=0.04; **Figure 4**). Three studies (25–27) were excluded from the meta-analysis as they did not report weight at the end of the study. However, these studies provided information on changes in BMI. The BMI of the intervention groups showed a significant decrease (MD: -2.42; 95% CI: -3.33 to -1.52; I^2^ = 35%; P<0.00001; **Figure 5**). One study (30) reported a significant impact on weight reduction in the intervention group. However, it should be noted that the intervention group also received oral weight loss medication in addition to behavioral interventions. Therefore, this study was not included in the current meta-analysis.

##### 3.4.1.2 Body mass index

**Figure 6:**
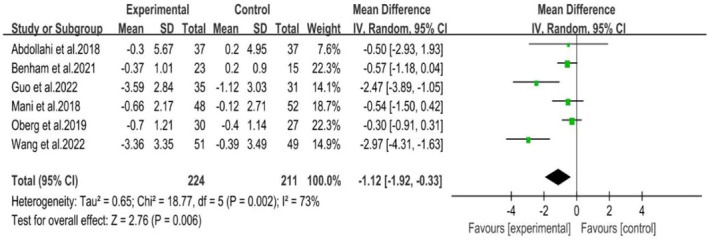
Forest plot of mean difference standardized of BMI.

BMI was reported in six RCTs (25–29, 31), including 224 participants in the intervention group and 211 participants in the control group. The random effects model was used for meta-analysis, and our results showed that behavioral intervention was associated with a significant decrease in BMI (MD: -1.12; 95% CI: -1.92 to -0.33; I^2^ = 73%; P=0.006; **Figure 6**).

Subgroup analyses were conducted based on random-effects models according to the specific method of behavioral interventions, including mobile health applications, supervised training, and encouragement courses, due to the high heterogeneity.

Based on the specific methods of behavioral intervention, there are two subgroups distinguished by the use of mobile health applications. Behavioral intervention was conducted through mobile health applications in two studies (26, 27). And the intervention group showed a significant decrease in BMI when compared to the control group (MD: -2.73; 95% CI: -3.71 to -1.76; I^2^ = 0%; P<0.00001; **Table 2**). However, supervised training and encouragement courses were used to modify behavior patterns in four studies (25, 28, 29, 31), but there was no significant difference in BMI between the groups (MD: -0.45; 95% CI: -0.84 to -2.29; I^2^ = 0%; P=0.02; **Table 2**).

**Table 2:** Subgroup analyses of BMI.

Subgroups	Study included number	Heterogeneity		Effect model	Meta analysis	
		P value	I^2^(%)		Relative effect (95%CI)	P value
Mobile health applications	2	0.62	0	Fixed	-2.73 (-3.71, -1.76)	<0.00001
Encouragement courses and supervision training	4	0.94	0	Fixed	-0.45 (-0.84, -2.29)	0.02

##### 3.4.1.3 Waist circumference

**Figure 7:**
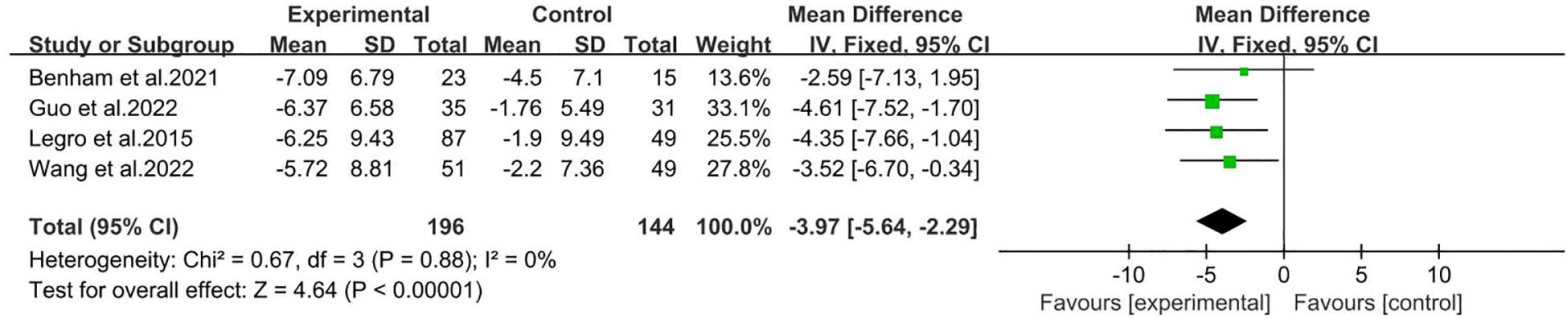
Forest plot of mean difference standardized of Waist circumference.

There were four studies reported Waist circumference (26, 27, 30, 31), including 196 participants in the intervention group and 144 participants in the control group. The results showed that behavioral intervention significantly improved waist circumference (MD: -3.97; 95% CI: -5.64 to -2.29; I^2^ = 0%; P<0.00001; **Figure 7**).

#### 3.4.2 Secondary outcomes

##### 3.4.2.1 Clinical parameters

###### 3.4.2.1.1 Psychological status: depression

Three studies (25, 26, 32)with a total of 118 participants in the intervention group and 99 in the control group displayed depression. The behavioral intervention group showed a significant change in depression (SMD: -1.12; 95% CI: -2.35 to -0.07; I^2^ = 92%; P=0.04; **Table 3**).

**Table 3:** Behavioral intervention compared with control for PCOS patients.

Outcome		Study included number	Heterogeneity		Effect model	Meta analysis	
			P value	I^2^		Relative effect (95%CI)	P value
**Anthropometric measurements**	Weight loss	4	1.00	0%	Fixed	-1.07 (-2.10, -0.03)	0.04
	Body mass index	6	0.002	73%	Random	-1.12 (-1.92, -0.33)	0.006
	Waist circumference	4	0.88	0%	Fixed	-3.97 (-5.64, -2.29)	<0.00001
**Clinical outcomes**	Depression	3	<0.00001	92%	Random	-1.21 (-2.35, -0.07)^a^	0.04
	Menstrual problems	3	0.93	0%	Fixed	0.17 (-0.11, 0.46)	0.23
	Hirsutism	3	0.16	46%	Fixed	-0.26 (-0.53, 0.00)	0.05
	Emotion	3	0.79	0%	Fixed	0.11 (-0.13, 0.36)	0.35
	Infertility	3	0.28	22%	Fixed	0.24 (-0.06, 0.54)	0.11
	Weight	2	0.40	0%	Fixed	0.58 (0.15, 1.02)	0.008
**Biochemical indexes**	High-density lipoprotein	2	0.08	67%	Random	0.03 (-0.12, 0.18)	0.69
	Low-density lipoprotein	2	0.76	0%	Fixed	-0.04 (-0.20, 0.12)	0.65
	Systolic blood pressure	2	0.49	0%	Fixed	1.31 (-2.36, 4.98)	0.48
	Diastolic blood pressure	2	0.42	0%	Fixed	-0.32 (-3.04, 2.40)	0.82
	HOMA-IR	3	0.61	0%	Fixed	-0.14 (-0.47, 0.19)	0.41
	Triglycerides	3	0.25	27%	Fixed	-0.16 (-0.27, -0.05)	0.004
	Total cholesterol	2	0.55	0%	Fixed	-0.04 (-0.23, 0.16)	0.71
	Testosterone	3	0.02	76%	Random	0.30 (-0.18, 0.78)^a^	0.22
	Fasting glucose	3	0.06	64%	Random	0.00 (-0.21, 0.21)	0.99
	Fasting insulin	3	0.41	0%	Fixed	-0.56 (-2.79, 1.68)	0.63
	HbA1C	2	0.95	0%	Fixed	-0.34 (-0.68, -0.00)^a^	0.05
	SHBG	2	0.63	0%	Fixed	0.05 (-0.27, 0.26)^a^	0.78

HOMA-IR, homeostasis model assessment as an index of insulin resistance; HbA1C, hemoglobin A1C; SHBG, sex hormone binding globulin; ^a^, standardized mean difference.

The meta-analysis revealed high heterogeneity among the studies. However, due to the limited number of available studies, it was not possible to explore the sources of heterogeneity through subgroup analysis.

###### 3.4.2.1.2 Quality of life

Overall, three literatures (28, 30, 32)assessed the quality of life, involving 161 patients supplemented with behavioral interventions and 103 patients in the control group. Quality of life was assessed by PCOSQ. There was no significant difference in the quality of life related to menstrual problems between the two groups (MD: 0.17; 95% CI: -0.11 to 0.46; I^2^ = 0%; P=0.23; **Table 3**). The similar results can also be observed in the hirsutism domain (MD: -0.26; 95% CI: -0.53 to 0.00; I^2^ = 46%; P=0.05; **Table 3**), the emotions domain (MD: 0.11; 95% CI: -0.13 to 0.36; I^2^ = 0%; P=0.35; **Table 3**), and the infertility problems domain (MD: 0.24; 95% CI: -0.06 to 0.54; I^2^ = 22%; P=0.11; **Table 3**). However, the results indicated that behavioral interventions had a significant positive impact on the quality of life of patients in terms of weight, as measured by the PCOSQ (MD: 0.58; 95% CI: 0.15 to 1.02; I^2^ = 0%; P=0.008; **Table 3**). One study (30) used diet pills in combination with behavioral interventions for weight loss was excluded from the analysis.

###### 3.4.2.1.3 Blood pressure

Only two RCTs have reported on blood pressure (28, 31). The behavioral intervention group did not show a significant difference in systolic blood pressure compared to the routine treatment group (MD: 1.31; 95% CI: -2.36 to 4.98; I^2^ = 0%; P=0.48; **Table 3**). Additionally, there was no significant difference in diastolic blood pressure between the two groups (MD: -0.32; 95% CI: -3.04 to 2.40; I^2^ = 0%; P=0.82; **Table 3**). Overall, the findings suggest that the behavioral intervention is not more effective than routine treatment in improving both systolic and diastolic blood pressure.

##### 3.4.2.2 Metabolic parameters

###### 3.4.2.2.1 Triglycerides

Three studies including 158 patients in intervention group and 111 patients in the control group provided data of TG (28, 30, 31). We observed a significant decrease in TG in the behavioral intervention group compared to the control group (MD: -0.16; 95% CI: -0.27 to -0.05; I^2^ = 27%; P=0.004; **Table 3**).

###### 3.4.2.2.2 HOMA–IR

There were three studies including 93 patients in intervention group and 89 patients in the control group reported HOMA–IR (28, 29, 31). The fixed effects model displayed no discernible difference between the intervention and control groups (MD: -0.14; 95% CI: -0.47 to 0.19; I^2^ = 0%; P=0.41; **Table 3**).

###### 3.4.2.2.3 Testosterone

Testosterone levels were mentioned in three studies (28–30), involving 171 participants in the intervention group and 131 in the control group. According to the random effects model, there was no statistically significant difference in testosterone levels between the two groups (SMD: 0.30; 95% CI: -0.18-0.78; I^2^ = 76%; P=0.22; **Table 3**). We found a high degree of heterogeneity, and further subgroup analysis was conducted according to the study duration. Two studies were conducted for four months, and the results showed a significant decrease in testosterone levels in the control group (SMD: 0.52; 95% CI: 0.15 to 0.89; I^2^ = 37%; P=0.005). Another study was conducted for one year found that behavioral intervention had no significant effect on testosterone levels. Further research is necessary for additional meta-analysis.

###### 3.4.2.2.4 Fasting glucose and fasting insulin

Three studies reported fasting glucose levels (28, 29, 31). The behavioral intervention group consisted of 101 patients, while the control group consisted of 94 patients. These studies also measured the fasting insulin levels, with 93 patients assigned to the behavioral intervention group and 89 patients assigned to the control group. Fasting glucose calculated using the random effects model. The analysis showed no statistically significant difference in fasting glucose between the two groups (MD: 0.00; 95% CI: -0.21 to 0.21; I^2^ = 64%; P=0.99; **Table 3**). A fixed-effects model was used to analyze fasting insulin levels, revealing no significant difference between the intervention and control groups (MD: -0.56; 95% CI: -2.79 to 1.68; I^2^ = 0%; P=0.63; **Table 3**).

###### 3.4.2.2.5 High-density lipoprotein and low-density lipoprotein

High-density lipoprotein and low-density lipoprotein were measured in two studies with study duration of six months and 12 months respectively (29, 32). The random effects model showed no significant difference in HDL levels between the behavioral intervention group and the control group (MD: 0.03; 95% CI: -0.12 to 0.18; I^2^ = 0%; P=0.69; **Table 3**). Fixed-effects modeling revealed no significant difference in LDL levels between the two groups (MD: -0.04; 95% CI: -0.20 to 0.12; I^2^ = 0%; P=0.65; **Table 3**).

###### 3.4.2.2.6 Total cholesterol

Two studies provided data on total cholesterol with 70 patients in intervention group and 66 patients in the control group (28, 31). In the fixed effects models, there was no significant difference in total cholesterol in the behavioral intervention group compared with the control group (MD: -0.04; 95% CI: -0.23 to 0.16; I^2^ = 0%; P=0.71; **Table 3**).

###### 3.4.2.2.7 Hemoglobin A_1C_

Only two studies reported HbA_1C_ levels (28, 31), which were evaluated using a fixed-effects model. The results showed no significant difference between the group that received the behavioral intervention and the control group (SMD: -0.34; 95% CI: -0.68 to -0.00; I^2^ = 0%; P=0.05; **Table 3**).

###### 3.4.2.2.8 Sex hormone binding globulin

Two studies mentioned SHBG (28, 29). One study reported significantly elevated SHBG levels (29), while the other study showed no clinically significant changes. More adequate data is needed for meta-analysis on this topic. Fixed effects model resulted that estradiol levels were not significantly different in two groups (SMD: 0.05; 95% CI: -0.27 to 0.26; I^2^ = 0%; P=0.78; **Table 3**).

In summary, we primarily found that behavioral interventions improved outcomes like weight loss, BMI, waist circumference, psychological status and TG. While other clinical manifestations and metabolic indexes were not significantly altered.

#### 3.4.3 Adverse reaction

The four studies (26, 29–31) included in the Meta-analysis mentioned adverse reactions. Two of the studies (26, 29) showed no adverse events. Adverse events were recorded in the remaining two studies, one (31) of which did not report trial-related adverse events, and the other (30) reported adverse events mainly related to specific other treatment modalities. Behavioral interventions may be a safe treatment for PCOS.

## 4 Discussion

In conclusion, lifestyle interventions recommended for patients with PCOS include exercise, adopting a balanced and nutritious dietary pattern, and behavioral changes (39). Furthermore, studies have demonstrated that lifestyle modifications are beneficial treatment methods for women with PCOS (20, 40, 41). Although there is some understanding of the effects of behavioral interventions on PCOS, only a few trials have confirmed these findings.

This study aimed to investigate the impact of different behavioral interventions on various aspects of health in patients diagnosed with PCOS. The study focused on analyzing the effects of these interventions on weight loss, BMI, waist circumference, clinical manifestations of PCOS and biochemical indicators. This meta-analysis included eight studies involving 744 reproductive-aged PCOS patients, we observed beneficial effect of behavioral interventions on various aspects of health in patients with PCOS, including weight loss, improvement in BMI, and reduction in waist circumference. This statement is consistent with Jiskoot et al.’s findings that behavioral interventions are crucial for achieving long-term weight loss and improving mental health (20, 42, 43). However, a meta-analysis report showed that cognitive-behavioral interventions alone did not have a significant effect on reducing weight in patients with PCOS (44). This difference may be related to the differences in study populations, intervention durations, and types of behavioral interventions.

In addition, our data also suggest that behavioral interventions can improve the patients’ quality of life in terms of depressive symptoms and weight. Previous studies in patients with PCOS have similarly reported that cognitive-behavioral interventions can reduce depressive scores in PCOS (35, 44, 45). Moreover, research has shown that using mobile applications and text messages as intervention measures can improve patients’ mental and physical health (22, 46), which is consistent with our study findings. Furthermore, in our study, subgroup analysis based on study duration revealed a significant decrease in testosterone in the control group. Possible reasons for this are the relatively high initial levels in the control group and the short duration of the study. More studies and longer follow-up time are needed to further clarify the effect of behavioral intervention on testosterone.

Our analysis suggests that behavior interventions through short message service (SMS), mobile health applications, supervised training, and encouragement courses can have beneficial impacts by inducing changes in patients’ behavior patterns. However, the effectiveness of behavior interventions in achieving other outcomes such as menstrual health, infertility, and emotional life quality has not been confirmed. Furthermore, there is insufficient evidence to support the effectiveness of behavior interventions in reducing systolic and diastolic blood pressure, HDL, LDL, HOMA-IR, T, Tch, TG, fasting glucose, fasting insulin, HbA_1C_, and SHBG. On the contrary, some studies have indicated that behavior interventions can have favorably affect patients’ menstrual cycles and fertility (47), which contradict our findings. This discrepancy may be attributed to the quality and quantity of included studies, and more definitive conclusions can be drawn through further relevant research.

Patients with PCOS have a significantly higher prevalence of overweight and obesity compared to non-PCOS patients (23). Most individuals with PCOS are overweight and obesity throughout their entire lifespan, and obesity exacerbates the reproductive, metabolic, and psychological symptoms of PCOS (48). Weight loss can bring about significant improvements in psychological symptoms (depression and quality of life), reproductive function (menstrual cycles and fertility), and metabolic symptoms (insulin resistance, metabolic syndrome, etc.) of patients, even if they remain in the overweight or obese range.

This meta-analysis has several advantages. It includes retrieval of multiple databases without any time restrictions. One of the strengths of our study is that it simultaneously investigates the common complications associated with PCOS patients, such as obesity, depression, and biochemical markers. Additionally, we also assessed the quality of life and blood pressure.However, there are several limitations to this study that must be considered when interpreting the results. The main limitation of this study is the limited number of published literatures evaluating the impact of behavioral interventions in patients with PCOS. Therefore, it was not possible to perform subgroup analysis based on all interventions and associated outcomes. Additionally, the original data of individual studies were not available and some studies were of low quality. Some trials lacked specific descriptions of whether behavioral interventions led to changes patients’ behavior, and some trials lasted less than 6 months, which is typically necessary for behavioral changes to occur in patients (49). Heterogeneity is a significant issue. There was significant heterogeneity in study participants, outcome measures, and intervention content, which could potentially affect the study results. Due to the limited number of studies and sample size, we were unable to conduct subgroup and sensitivity analyses to explore the sources of heterogeneity. Furthermore, the use of a self-reported questionnaire to assess outcome may have introduced bias in some studies. In the future, more well-designed clinical trials are needed to investigate the effects of behavioral interventions on PCOS patients. Long-term follow-up is also necessary to observe the long-term effectiveness of behavioral interventions in PCOS patients.

## 5 Conclusion

Our analysis indicates that using interventions such as text messages, mobile health applications, supervised training, and encouragement courses can improve weight loss, BMI, waist circumference, and depressive symptoms in patients with PCOS. However, because the intervention duration was short and there was no long-term follow-up, it is not possible to determine the long-term benefits for patients. Therefore, further well-designed studies are still needed to clarify and confirm the effects of behavioral interventions in patients with PCOS.

## Data availability statement

The original contributions presented in the study are included in the article/**Supplementary Material**. Further inquiries can be directed to the corresponding author.

## Author contributions

MX: Data curation, Formal analysis, Writing – original draft. YY: Data curation, Formal analysis, Writing – review & editing. JZ: Conceptualization, Supervision, Validation, Writing – review & editing.
