# Supplementary material for: The effects of behavioral intervention on anthropometric, clinical, and biochemical parameters in patients with polycystic ovary syndrome: a systematic review and meta-analysis
Source: Front Endocrinol (Lausanne). 2024 Feb 28;15:1297841. doi: 10.3389/fendo.2024.1297841 (PMC10933019; doi:10.3389/fendo.2024.1297841)
Supplement: Supplementary file 1 [file DataSheet_1.doc]

**Supplementary Information**

**Search strategy**

**Pubmed:**

1# （Polycystic Ovary Syndrome OR Ovary Syndrome, Polycystic OR Syndrome, Polycystic Ovary OR Polycystic ovary disease OR Stein-Leventhal Syndrome OR Stein Leventhal Syndrome OR Syndrome, Stein-Leventhal OR Sclerocystic Ovarian Degeneration OR Ovarian Degeneration, Sclerocystic OR Sclerocystic Ovary Syndrome OR Polycystic Ovarian Syndrome OR Ovarian Syndrome, Polycystic OR Polycystic Ovary Syndrome 1 OR Sclerocystic Ovaries OR Ovary, Sclerocystic OR Sclerocystic Ovary OR hyperandrogenism OR Hypertrichosis OR Hirsutism OR "PCOS" OR "PCO" OR "PCO-S"）

2# （behavioral therapy OR behavioral modification OR behavioral intervention OR Cognitive Behavioral Therapy OR behavior change intervention OR Intervention OR interventions OR psychotherapy OR psycho-therapy OR cognitive therapy OR educational-therapy OR online-therapy OR training OR remediation OR behavior）

3# （Randomized Controlled Trial[ptyp] AND "humans"[MeSH Terms]）

4# ( 1# AND 2#) AND 3#

((Polycystic Ovary Syndrome OR Ovary Syndrome, Polycystic OR Syndrome, Polycystic Ovary OR Polycystic ovary disease OR Stein-Leventhal Syndrome OR Stein Leventhal Syndrome OR Syndrome, Stein-Leventhal OR Sclerocystic Ovarian Degeneration OR Ovarian Degeneration, Sclerocystic OR Sclerocystic Ovary Syndrome OR Polycystic Ovarian Syndrome OR Ovarian Syndrome, Polycystic OR Polycystic Ovary Syndrome 1 OR Sclerocystic Ovaries OR Ovary, Sclerocystic OR Sclerocystic Ovary OR hyperandrogenism OR Hypertrichosis OR Hirsutism OR "PCOS" OR "PCO" OR "PCO-S") AND(("behavior therapy"[MeSH Terms] OR behavioral modification OR behavioral intervention OR Cognitive Behavioral Therapy OR behavior change intervention OR educational-therapy OR online-therapy OR training OR remediation OR behavior)))AND (Randomized Controlled Trial[ptyp] AND "humans"[MeSH Terms])

**Cochrane:**

## 264 Trials matching (Polycystic Ovary Syndrome OR Ovary Syndrome, Polycystic OR Syndrome, Polycystic Ovary OR Polycystic ovary disease OR Stein-Leventhal Syndrome OR Stein Leventhal Syndrome OR Syndrome, Stein-Leventhal OR Sclerocystic Ovarian Degeneration OR Ovarian Degeneration, Sclerocystic OR Sclerocystic Ovary Syndrome OR Polycystic Ovarian Syndrome OR Ovarian Syndrome, Polycystic OR Polycystic Ovary Syndrome 1 OR Sclerocystic Ovaries OR Ovary, Sclerocystic OR Sclerocystic Ovary OR hyperandrogenism OR Hypertrichosis OR Hirsutism OR "PCOS" OR "PCO" OR "PCO-S") AND(behavior therapy OR behavioral modification OR behavioral intervention OR Cognitive Behavioral Therapy OR behavior change intervention OR educational-therapy OR online-therapy OR training OR remediation OR behavior*) in Title Abstract Keyword AND (randomized controlled trial or (trial* or random* or RCT*)) in Title Abstract Keyword

**Medline:**

TX ((Polycystic Ovary Syndrome OR Ovary Syndrome, Polycystic OR Syndrome, Polycystic Ovary OR Polycystic ovary disease OR Stein-Leventhal Syndrome OR Stein Leventhal Syndrome OR Syndrome, Stein-Leventhal OR Sclerocystic Ovarian Degeneration OR Ovarian Degeneration, Sclerocystic OR Sclerocystic Ovary Syndrome OR Polycystic Ovarian Syndrome OR Ovarian Syndrome, Polycystic OR Polycystic Ovary Syndrome OR Sclerocystic Ovaries OR Ovary, Sclerocystic OR Sclerocystic Ovary OR hyperandrogenism OR Hypertrichosis OR Hirsutism OR "PCOS" OR "PCO" OR "PCO-S")) AND TX ((behavior therapy OR behavioral modification OR behavioral intervention OR Cognitive Behavioral Therapy OR behavior change intervention OR educational-therapy OR online-therapy OR training OR remediation)) AND TX ( randomized controlled trials or rtc or randomised control trials )

**Embase**:

1# 'polycystic ovary syndrome'/exp OR 'polycystic ovar$':ti,ab,kw OR 'sclerocystic adj3 ovar$':ti,ab,kw OR 'stein leventhal':ti,ab,kw

2# 'behavior therapy'/exp OR 'behavioral modification':ti,ab,kw OR 'behavioral intervention':ti,ab,kw OR 'cognitive behavioral therapy':ti,ab,kw OR 'behavior change intervention':ti,ab,kw OR 'educational therapy':ti,ab,kw OR 'online therapy':ti,ab,kw OR training:ti,ab,kw OR remediation:ti,ab,kw

3# 1# and 2#

4# 'randomized controlled trial':ti,ab,kw OR trial*:ti,ab,kw OR random*:ti,ab,kw OR rct*:ti,ab,kw

5# 3# and 4#
